# Spatial and Temporal Dynamics of Electrical and Photosynthetic Activity and the Content of Phytohormones Induced by Local Stimulation of Pea Plants

**DOI:** 10.3390/plants9101364

**Published:** 2020-10-15

**Authors:** Maria Ladeynova, Maxim Mudrilov, Ekaterina Berezina, Dmitry Kior, Marina Grinberg, Anna Brilkina, Vladimir Sukhov, Vladimir Vodeneev

**Affiliations:** 1 Department of Biophysics, National Research Lobachevsky State University of Nizhny Novgorod, 23 Gagarin Avenue, 603950 Nizhny Novgorod, Russia; ladeynova.m@yandex.ru (M.L.); mtengri@yandex.ru (M.M.); berezina.kat@gmail.com (E.B.); dimakior@mail.ru (D.K.); mag1355@yandex.ru (M.G.); annbril@mail.ru (A.B.); vssuh@mail.ru (V.S.); 2 Earth’s Electromagnetic Environment Laboratory, Institute of Applied Physics, 46 Ulyanova Street, 603950 Nizhny Novgorod, Russia

**Keywords:** abiotic stress, electrical signal, photosynthesis, phytohormones, *Pisum sativum*

## Abstract

A local leaf burning causes variation potential (VP) propagation, a decrease in photosynthesis activity, and changes in the content of phytohormones in unstimulated leaves in pea plants. The VP-induced photosynthesis response develops in two phases: fast inactivation and long-term inactivation. Along with a decrease in photosynthetic activity, there is a transpiration suppression in unstimulated pea leaves, which corresponds to the long-term phase of photosynthesis response. Phytohormone level analysis showed an increase in the concentration of jasmonic acid (JA) preceding a transpiration suppression and a long-term phase of the photosynthesis response. Analysis of the spatial and temporal dynamics of electrical signals, phytohormone levels, photosynthesis, and transpiration activity showed the most pronounced changes in the more distant leaf from the area of local stimulation. The established features are related to the architecture of the vascular bundles in the pea stem.

## 1. Introduction

Plants are exposed to various adverse environmental factors, acting in local or systemic way. Many local stressors, such as mechanical damage, excessive light, and high and low temperatures, cause the activation of defense reactions not only at the site of action, but also at a considerable distance in non-stressed parts of the plant. The development of systemic adaptation requires the generation and propagation of stress signals [1], among which chemical, hydraulic, and electrical signals are distinguished in plants [2]. Electrical signals include three main types: action potential (AP) induced by non-damaging stimuli, variation potential (VP) (otherwise called slow wave potential (SWP)) induced by local damage or heating, and systemic potential (SP) induced by chemical factors or damage [2,3]. VP is a transient change in membrane potential with a relatively fast depolarization phase and a long-term and variable repolarization phase. VP propagates over long distances at a speed from several cm/min to tens of cm/min from the area of stimulation to intact plant areas, where it can induce a functional response [4,5]. Responses induced by local stimuli include an increase in the ATP concentration [6], changes in the activity of photosynthesis [5] and transpiration [7], activation of respiration [6,8], suppression of phloem transport [9], activation of the expression of protective genes [10,11,12], genes that determine the properties of cellular walls [13], and others.

The best studied VP-induced photosynthetic response is a transient decrease in the activity of photosynthetic processes [4,14,15,16]. The photosynthetic response can have different dynamics, including fast (from several min to several tens of min after stimulation) and long-term (from tens of min to several hours after stimulation) phases [5,17]. The characteristics of the photosynthetic response vary in different plant species, show dependence on age and type of stimulus [18,19,20]. Thus, in tobacco [17] and pumpkin [21] plants, the photosynthetic response is represented only by long-term inactivation, while in other plants (e.g., wheat, pea, tomato, sundew), the response includes both fast and long-term inactivation of photosynthesis.

The development of a photosynthetic response in unstimulated parts of the plant is a consequence of the propagation of a distant stress signal. At the same time, apparently, the mechanisms of induction of fast and long-term phases of photosynthetic responses are different. To date, the mechanism of the development of the first fast phase of the photosynthetic response has been well studied. Rapid transient inactivation of photosynthesis is caused by pH shifts—temporary acidification of the cytoplasm and alkalization of the apoplast, which occur due to the temporary inhibition of H^+^-ATPase activity during VP generation [14,22]. Such changes in pH lead to a decrease in CO_2_ transport into cells, a decrease in the activity of enzymes of the Calvin cycle, and an increase in heat loss of energy of absorbed light, resulting in inactivation of photosynthesis [5].

The long-term phase of the photosynthetic response appears to have a different induction mechanism. In particular, the participation of stress phytohormones, such as abscisic acid (ABA) and jasmonic acid (JA) [16,23], has been suggested. However, the available information on the role of these hormones in various plants in the induction of photosynthetic responses under local action of stressors is rather contradictory. Thus, in tobacco plants, a local burn causes a rapid increase in the ABA concentration and a later increase in the JA concentration in an unwounded leaf, which indicates a possible role for ABA in stomatal closure and a decrease in the rate of CO_2_ assimilation at the initial stage, while JA induces a later phase of the response [17]. In tomato plants, changes in the ABA concentration in unwounded leaves were not registered, while the systemic JA concentration was significantly increased. In this case, however, the initial level of ABA was important, which determines the nature of the differences in gas exchange [23]. In the trap of the insectivorous plant of the sundew *Drosera capensis*, local wounding causes an increase in the concentration of jasmonates and ABA along with a decrease in the activity of photosynthesis [24]. At the same time, the role of jasmonates in the induction of photosynthesis responses was suggested, since the most significant suppression of photosynthesis was observed when jasmonate reached its maximum concentrations, and, on the contrary, the photosynthetic activity was restored as the concentration of jasmonate decreased. In general, the small amount of available experimental data and their variability do not allow making an unambiguous conclusion about the role of a certain hormone in the induction of a certain phase of the photosynthetic response. It should also be noted that the spatial dynamics of both the local stimulus-induced photosynthesis responses and the hormone concentration are insufficiently studied. In particular, there is information about the registration of the dynamics and response of photosynthesis and the concentration of phytohormones only in the leaf adjacent to the stimulated leaf; there is no information on the responses in more distant leaves [23].

The aim of this work was to study the spatiotemporal dynamics of the local stimulation-induced electrical signals and photosynthesis response and the content of JA and ABA in unstimulated leaves of pea.

## 2. Results

### 2.1. Electrical Signals Induced by Local Stimulation

**Figure 1:**
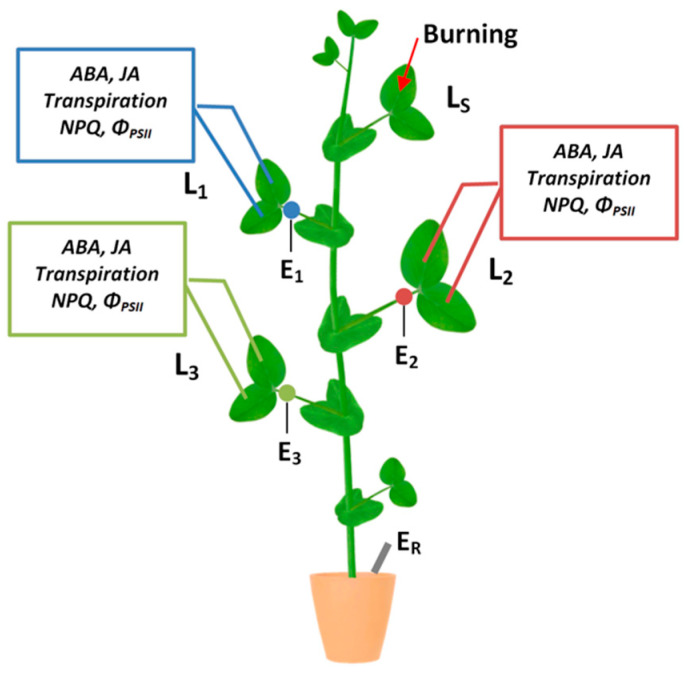
Scheme of the experiment: local stimulation by burning tip of the upper fully developed leaf (L_S_), recording of electrical signals, measurement of photosynthetic parameters, transpiration intensity measurement, quantification of phytohormones in unstimulated leaves (L_1_, L_2_, L_3_). E_1_, E_2_, and E_3_ are electrodes, which were placed on the petioles of the first, second and third leaves, respectively; E_R_ is the reference electrode.

**Figure 2:**
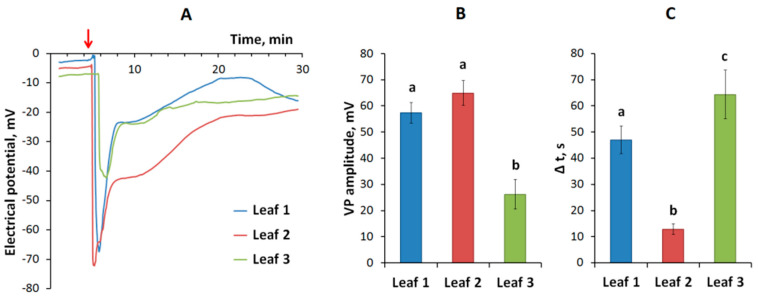
Variation potential (VP) induced by local burning of a pea leaf: (**A**) the typical record of electrical reaction; (**B**) VP amplitude in the first, second and third leaves, located below the stimulated leaf; (**C**) the time interval between stimulation and the VP occurrence in the first, second and third leaves. (**A**): the red arrow indicates the moment of stimulation. (**B**,**C**): different letters denote significant differences between columns, *p* < 0.05.

The burning of a pea leaf (Figure 1) causes a propagation of VP in unstimulated leaves (Figure 2A). The VP amplitude on the first leaf, located below the stimulated leaf, is 57 ± 4 mV, on the second leaf—65 ± 5 mV, on the third leaf—26 ± 6 mV (Figure 2B). It should be noted that the reaction on the second leaf, which is more distant from the local stimulation area, occurs earlier than on the first leaf, located closer to the stimulation area. The time interval between stimulation and the VP occurrence in the first, second and third leaves is 47, 13, and 64 s, respectively (Figure 2C).

### 2.2. Changes in Photosynthetic Activity Induced by Local Stimulation

**Figure 3:**
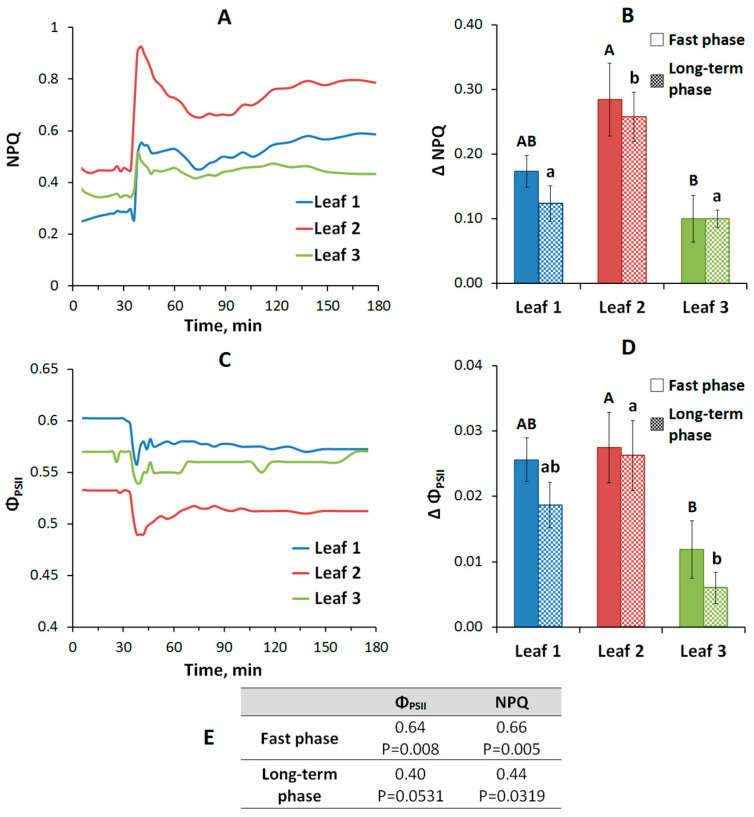
Changes in photosynthesis activity induced by variation potential (VP) in the first, second and third leaves, located below the stimulated leaf: (**A**) the typical records of non-photochemical quenching (NPQ) changes in the first, second and third leaves; (**B**) amplitudes of NPQ changes in the first, second and third leaves; (**C**) the typical records of Φ_PSII_ changes in the first, second and third leaves; (**D**) amplitudes of Φ_PSII_ changes in the first, second and third leaves; (**E**) correlations between the amplitude of the photosynthesis response and the VP amplitude. Correlations were determined for simultaneous recording of electrical signals and photosynthesis responses on separate plants (n = 8). Different uppercase letters denote significant differences between columns of fast phase, different lowercase letters denote significant differences between columns of long-term phase, *p* < 0.05.

Local burning causes a transient decrease in the activity of photosynthesis in unstimulated leaves, which is manifested in a decrease in the level of efficiency of photochemical reactions of photosystem II (Φ_PSII_) and an increase in the level of non-photochemical quenching (NPQ) (Figure 3). The initial values of NPQ were 0.34 ± 0.01; 0.41 ± 0.01; 0.39 ± 0.02; the initial values of Φ_PSII_ were 0.58± 0.02; 0.54 ± 0.01; 0.54 ± 0.01 in the first, second and third leaves, respectively. A decrease in photosynthesis activity develops in two phases (Figure 3). The first fast wave of a decrease in photosynthesis activity starts in a few min after the VP occurrence in the leaf and lasts about 20 min; the second longer wave begins to develop at the end of the first wave and lasts over an hour. The amplitudes of the first wave of NPQ and Φ_PSII_ do not show significant differences between the first and second leaves, only there is a trend towards a greater amplitude in the second leaf (Figure 3), in the third leaf the amplitude of the photosynthesis response is statistically significantly lower. The amplitude of the second long-term wave of photosynthetic response in the second leaf located below the stimulated leaf exceeds those in the first and third leaves (Figure 3). In general, it is necessary to note a greater amplitude of the photosynthetic response in the second leaf, which is more expressed for the long-term wave.

### 2.3. Changes in Transpiration Intensity Induced by Local Stimulation

**Figure 4:**
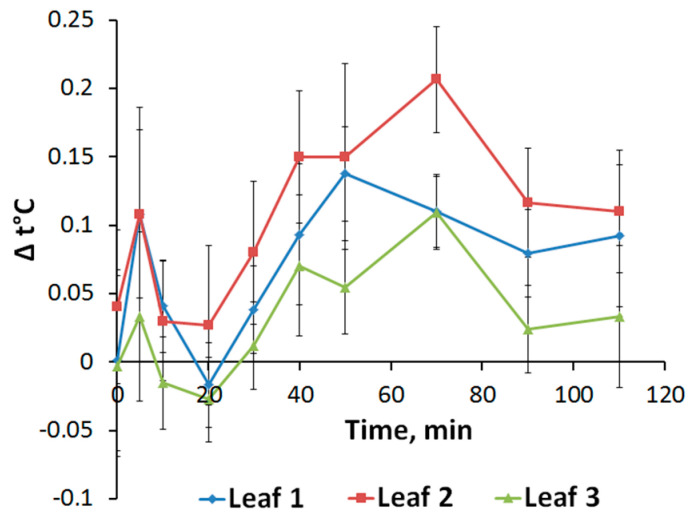
Dynamics of leaf temperature induced by local burning. The moment of stimulation corresponds to the point of 0 min. ∆t is the temperature difference between the corresponding leaves of stimulated and unstimulated plants.

The changes in transpiration caused by electrical signals were assessed by the change in the leaf temperature measured with a thermal imager, which made it possible to determine the spatio-temporal dynamics of the studied parameter in all investigated leaves. Local burning causes a transient increase in the temperature of unstimulated leaves, which indicates a reducing transpiration intensity (Figure 4). The rise in leaf temperature begins about 20 min after stimulation, reaching a maximum at 50–70 min, returning to close to the initial values after about 2 h. The temperature rise is slightly higher for the second leaf compared to the first. Temperature changes in the third leaf are less expressed. Taking into account the close relationship of temperature changes with the process of plant leaf transpiration [25,26], it can be assumed that the revealed responses reflect stomatal closure related to the propagation of electrical signals, which has been shown in a number of works on various plants [17,18,23,27], including pea [6,7]. This is also confirmed by the high similarity of the dynamics of temperature and gas-exchange during simultaneous recording (Figure S1).

### 2.4. Dynamics of Abscisic Acid and Jasmonic Acid Content Induced by Local Stimulation

**Figure 5:**
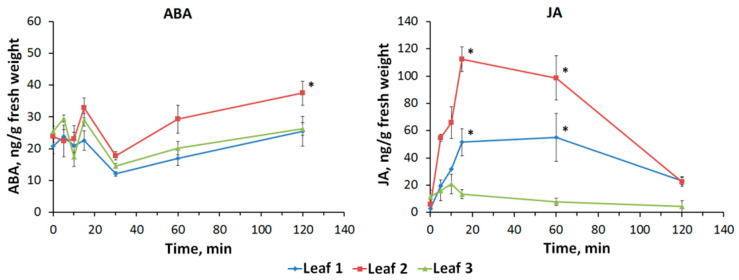
Time course of changes in the phytohormones abscisic acid (ABA) and jasmonic acid (JA) induced by local burning. The moment of stimulation corresponds to the point of 0 min. * indicates that difference from control value (0 min) were significant (*p* < 0.05).

Local burning causes a transient increase in ABA and JA concentrations in unstimulated leaves (Figure 5). There are insignificant fluctuations in the ABA concentration: only 120 min after the burning, there is a statistically significant increase in the second leaf. In the first and third leaves, the ABA concentration remains relatively constant with small fluctuations. Considering the dynamics of the JA content, one can observe a fast increase in the concentration already 5 min after the burning. JA concentration reaches its maximum 15 min after the burning, and then a gradual decrease begins throughout investigated time interval. The greatest changes in concentration are observed in the second leaf, a smaller amplitude of changes in JA concentration is typical for the first leaf, in the third leaf there are no significant changes in JA concentration.

### 2.5. Ways of Long-Distance Signal Propagation in Pisum Sativum Stem

**Figure 6:**
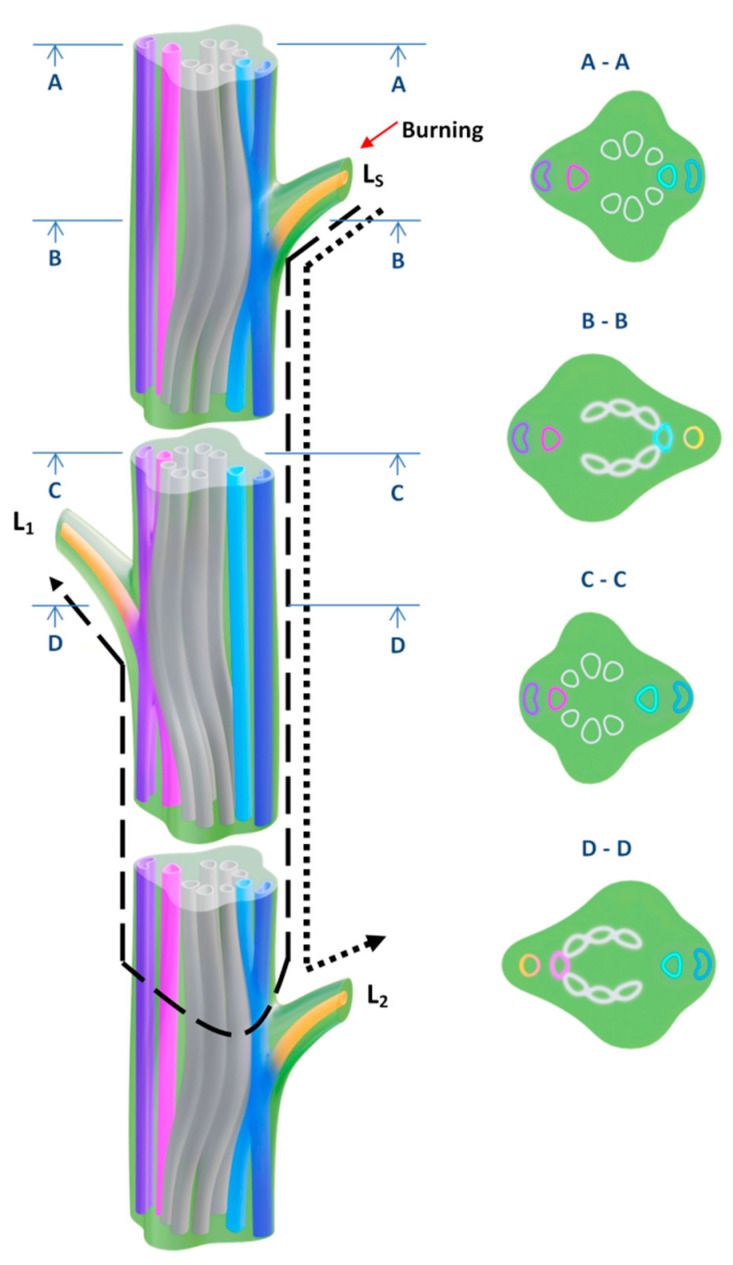
Hypothetical scheme of long-distance signal propagation. Three-dimensional diagram of the vascular bundles in the stem of Pisum sativum and schematic cross sections of internodes and nodes. All the changes in the vascular bundle system occur at the nodal region and this is the reason for omitting the internodes in the figure. Dotted line indicates the pathway of long-distance signal from the stimulated leaf (L_S_) to the second unstimulated leaf (L_2_). Dashed line indicates the pathway of long-distance signal from the stimulated leaf (L_S_) to the first unstimulated leaf (L_1_).

The observed features of electrical and hormonal signals propagation and subsequent functional response required an analysis of the signal propagation ways, which we performed on the basis of the reconstruction of the three-dimensional structure of the vascular bundles in the stem of *Pisum sativum* (Figure 6). Pea stem in a cross section represents a tetragon. In the internodes, central cylinder consists of separate bundles (colored in gray on the scheme). Bundles in opposite angles enter below or above located leaves. Because of pea alternate phyllotaxis, angle bundles from one side enter only even leaves (for example, the second and the fourth leaves; colored in blue) while angle bundles from the opposite side enter only uneven leaves (for example, the first and the third leaves; colored in purple). Close to node, angle bundles from the node side, where leaf forms (purple), and central bundles (gray) merge in a horseshoe-like structure. It is necessary to note, as for angle bundles related to leaves from the opposite side (blue) they remain separate. Leaving the node bundles partially recombine and in internode horseshoe-like structure disappears. In the next node central bundles (gray) merge with another angle bundles (blue).

**Figure 7:**
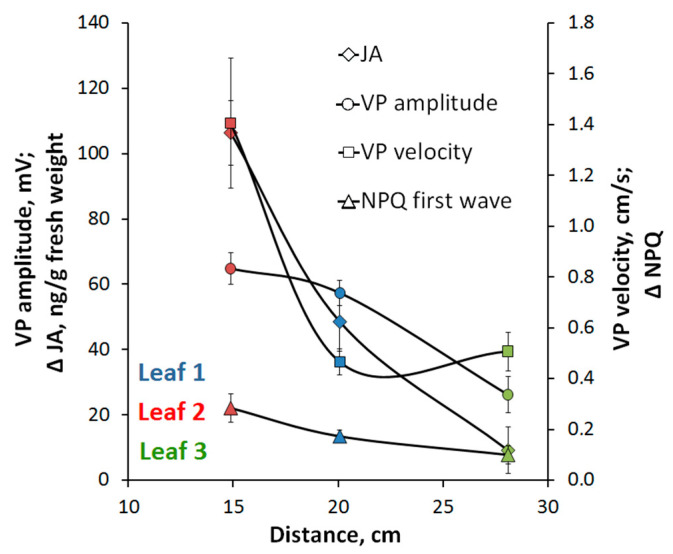
Dependence of the VP amplitude, VP velocity, the amplitude of the hormonal response and the photosynthesis response on the distance from the area of local stimulation according to the hypothetical scheme of long-distance signal propagation.

In pea externally measured distances between the stimulated leaf and the first, the second, and the third leaves amount to 10.9 ± 1.1 cm, 15.2 ± 1.2 cm, and 19.7 ± 1.5 cm, respectively. Since the VP propagation is associated with vascular bundles, in particular with xylem [3,28], VP-covered distances differ from the externally measured distances. According to the scheme (Figure 6) signal-covered distances (pointed with arrows on the scheme) amount to ~20 cm, 15 cm, and 28 cm for the first, the second, and the third leaves, respectively. Relation between VP amplitudes and signal-covered distances (Figure 7) clearly shows VP amplitude decrease with distance increase. The same tendencies are observed for decrease of hormonal signal and photosynthetic response (Figure 7).

## 3. Discussion

Our results show that the local burning the upper leaf induces propagation of the electrical signal (VP), influences content of phytohormones (ABA and JA) and changes in photosynthesis and transpiration in other leaves, which are not burned. Revealed VP properties are similar to ones, which were preliminarily shown in peas [6,22,29,30] and in other higher plants [2]. It is known that the VP propagation can be related to propagation of the hydraulic wave and/or chemical signal (maybe, ROS) from the damaged zone [3,31,32]. The followed generation of VP is probable to be caused by activation of ROS-dependent [32,33] or mechanosensitive [34] calcium channels and the Ca^2+^ influx [3], which induces the transient inactivation of the H^+^-ATPase in the plasma membrane.

Along with the changes in electrical activity, a local burning causes a change in the content of phytohormones in unstimulated leaves of pea (Figure 5). It can be assumed that the induction of systemic production of phytohormones is related to the propagation of an electrical signal. This assumption is presented in a number of works that include simultaneous registration of electrical signals and hormone concentration under the action of local stimuli [11,17,23,24,35,36,37]. Changes in concentrations of Ca^2+^, H^+^ and ROS, which accompany to VP, are the most likely mechanism of changes in phytohormone production. In particular, Fisahn et al. [38] showed that the Ca^2+^ influx was necessary for induction of increase of the JA concentration after propagation of heating-induced electrical signals in potato. Moreover, stimulation of the JA production in *Arabidopsis* required increase of the cytoplasm Ca^2+^ concentration, which was related to changes in activity of NADPH-oxidase and H_2_O_2_ production [39]. Finally, fluctuation in the intracellular Ca^2+^ concentration can be a linker between the ROS increase and the changes in ABA and JA concentrations under action of local stressor [31,35,40]. It should be additionally noted that changes in activity of the plasma membrane H^+^-ATPase can also influence the JA concentration [36].

The Ca^2+^-induced increase of concentrations of phytohormones can be related to influence of calcium ions on activities of enzymes, which participate in production of these phytohormones. In particular, Ca^2+^ can activate enzyme LOX6, which participates in the JA synthesis [41]. Additionally, stimulation of the JA production after mechanical damage [42] can be caused changes in activity of the phospholipase D [43]; it is known that this enzyme is activated by Ca^2+^ influx [44]. Ways of influence of other signal molecules (ROS, H^+^) on the phytohormones production are not clear; however, their effects can be potentially related to interactions between Ca^2+^, ROS and pH signal ways [45].

Thus, it can be hypothesized that electrical signals (VP) and changes in Ca^2+^, H^+^ and ROS, which accompany to the VP, are probably to cause changes in concentrations of investigated phytohormones (mainly, JA) after local burning. The presence of potential ways of influencing VP on the production of stress phytohormones, discussed above, as well as the short time interval between the stimulus and initiation of increase of the JA concentration in unstimulated leaves (Figure 5), may indicate that an increase of JA concentration is related to its production in unstimulated leaves due to the VP propagation. At the same time, there are works which show that transport of phytohormones from the stimulated zone can have high velocity [46,47,48]; as a result, we cannot fully exclude independent propagation of investigated phytohormones from the burned zone.

It is probable that the VP-induced increase of the JA concentration can participate in forming of the electrical signals-induced photosynthetic response. Our results show (Figure 3) that VP induced two-stage photosynthetic response. The fast photosynthetic inactivation is probable to be caused by increase of the extracellular pH and decrease of the intracellular pH, which accompany to the VP generation [14,15,22]. The relation between the fast photosynthetic inactivation and processes of the VP generation is supported by relatively high correlations of magnitudes of changes in Φ_PSII_ and NPQ with VP amplitude (Figure 3). However, these correlation coefficients are lower for the long-term photosynthetic inactivation (Figure 3); the result supports other mechanisms of this inactivation.

The long-term photosynthetic inactivation [15,17,23] can be caused by stimulation of JA production, which we observed in unstimulated leaves (Figure 5). JA concentration increase is reached before development of the long-term photosynthetic inactivation (but after the fast inactivation) (Figure 3 and Figure 5). Furthermore, magnitudes of changes in the JA concentration and photosynthetic changes in different leaves seem to be similar (Figure 3 and Figure 5). Moreover, recorded changes in the leaf temperature reflect VP-induced transpiration reduction (Figure 4) and, probably, stomata closing; magnitudes of this effect in different leaves are similar with magnitudes of changes in the JA concentration or magnitudes of the long-term photosynthetic inactivation (Figure 3 and Figure 5). Considering the strong relation of the magnitude of the long-term photosynthetic inactivation to the transpiration rate in peas [7], it can be supposed that the JA-induced stomata closing can be important mechanism of the long-term inactivation of photosynthesis. The hypothesis is in a good accordance with literature data about influence of JA on the stomata [49,50]; in particular, increase of the JA concentration in the guard cells decreases stomata opening for 5–15 min after this increase. It is important that the JA influence can be both independent and related to ABA pathways [50,51].

Another important result of our work is the revealing of paradoxical dependence of parameters electrical signals, photosynthetic and transpiration responses and changes in the phytohormones production on distance from the burned zone (Figure 2B,C, Figure 3, Figure 4 and Figure 5). The responses in the second leaf are initiated earlier than ones in the first leaf (which is nearest to the burned zone); magnitudes of responses in the second leaf are also maximal. It is probable that this paradoxical dependence is related to specific architecture of the vascular bundles in peas. In particular, it is known [52,53] that connection between the nearest leaves through the vascular bundles is absent; this connection is observed for leaves in the same side of the stem (i.e., the first leaf is directly connected with the third leaf, the second leaf is directly connected with the fourth leaf, etc.). Our anatomical analysis supports this connection between leaves in peas and shows that the real distance from the burned zone (length of the vascular bundles) can be strongly differed from this distance on the stem (Figure 7).

Propagation of the electrical signals in plants is known to be mainly related to the vascular bundles [2,3,4], in particular to xylem [3,28]; it can be expected that parameters of VP and investigated physiological responses should be dependent on lengths of the vascular bundles between the burned and analyzed zones. Analysis of dependence of investigated parameters on these lengths (Figure 7) supports this supposition: the typical dependences of parameters of signals and responses on distance from the burned zone are observed. It should be noted that our results are in a good accordance with results of other works; e.g., VP is only observed on the stimulated side of the stem in sunflower [54] or electrical and JA signals are mainly transmitted into leaves, which are directly connected by the vascular bundles with the stimulated leaf [11,55].

As whole, we can hypothesize the followed chain of events participating in forming of the photosynthetic response in unstimulated leaf of pea plants after the local action of stressors. The local damage induces the VP propagation, which is accompanied to changes in concentrations of Ca^2+^, H^+^, and ROS. The VP-related changes in pH induce the fast photosynthetic inactivation; which is analyzed in details in our earlier work [5]. Alternatively, VP-related changes in concentrations of Ca^2+^, H^+^ and ROS induces increase of the JA concentrations in non-damaged leaves; the increased JA concentrations decreases stomata opening and, thereby, induced the long-term photosynthetic inactivation.

## 4. Materials and Methods

### 4.1. Plant Material

Pea (*Pisum sativum* L.) seedlings were hydroponically cultivated in Binder KBW 720 growth chamber (Binder GmbH, Tuttlingen, Germany) at 24 °C, 16/8 h photoperiod. For the experiments, 19 day old plants with 5 fully developed leaves were used.

### 4.2. Electrical Activity Measurement

Electrical signals were extracellularly measured with Ag^+^/AgCl electrodes EVL-1MZ (Gomel Plant of Measuring Devices, Gomel, Belarus). The electrodes were connected with high-impedance amplifier IPL-113 (Semico, Novosibirsk, Russia) and a personal computer. Measuring electrodes were set in contact with a plant by cotton threads wetted with standard solution (1 mM KCl, 0.5 mM CaCl_2_, 0.1 mM NaCl). Three measuring electrodes were arranged on petioles near leaflets of three leaves below the stimulated leaf (Figure 1). The reference electrode was set in standard solution that covered plant roots. Measurements were performed on plants acclimated to measuring system for 1.5 h. Variation potential generation was induced by burning tip of the upper fully developed leaf with an open flame for 2–3 s.

### 4.3. Photosynthetic Activity Measurement

Spatio-temporal variations in the PSII chlorophyll fluorescence were assessed using Imaging pulse-amplitude modulation (PAM) chlorophyll fluorometer Open FluorCam FC 800-O/1010 (Photon Systems Instruments, Drasov, Czech Republic).

Plant was set in 20 × 20 cm measuring part of fluorometer and acclimated to measuring system for 1.5 h. After dark adaptation (15 min), red actinic light (280 μmol m^−2^ s^−1^, 617 nm) was switched on for 30 min. Then saturated light impulses (4000 μmol m^−2^ s^−1^, cool white light 6500 K, 800 ms) were given. The measuring light impulses −617 nm, 20 ms.

F, F_m_’ and F_m_ were measured and photochemical quantum yield of PSII (Φ_PSII_) and non-photochemical quenching (NPQ) as parameters that reflect photosynthetic light reactions intensity were calculated. Φ_PSII_ was calculated using the equation

(1) $$\Phi_{\mathrm{PSII}}{=}^{\;}\frac{F_{m}\prime -F}{F_{m}\prime }$$

where F and F_m_’ are current and maximum yield of PSII chlorophyll fluorescence under light conditions.

NPQ was calculated using the equation

(2) $$\mathrm{NPQ}{=}^{\;}\frac{F_{m}-{\;F}_{m}\prime }{F_{m}\prime }$$

where F_m_ is maximum yield of PSII chlorophyll fluorescence [56].

Leaf photosynthetic activity was recorded simultaneously with electrical activity on petioles of the same leaves (Figure 1).

### 4.4. Transpiration Intensity Measurement

Spatial distribution of changes in transpiration was assessed by leaf thermal imaging [25,26] with Testo 885 imager (Testo, Lenzkirch, Germany). Thermal images were taken together on control and stimulated plants planted in different vessels. Image analysis was performed with Testo “IRSoft” software. The difference between leaf temperature of stimulated plant and of control one reflected changes in transpiration.

### 4.5. HPLC-MS Analysis of Phytohormones

Phytohormones content was measured in three leaves below the stimulated leaf (Figure 1). After leaf burning leaflets of the first, second and third leaves were harvested at 0, 5, 10, 15, 30, 60, and 120 min. Harvested leaves were immediately weighted, frozen in liquid nitrogen, ground into powder with mortar and pestle and extracted with extraction solution (MeOH:H_2_O:HCOOH = 80:19:1) containing 1 ng/mL [^2^H]_6_ABA (Olchemim, Olomouc, Czech Republic) as internal standard. Extraction was performed in 1:20 ratio for 18 h at 4 °C using Multi Bio RS-24 rotator (Biosan, Riga, Latvia). After centrifugation on Z 36 HK centrifuge (Hermle Labortechnik, Wehingen, Germany) at 4 °C for 25 min, 20,040 g, supernatant was collected and the residue was extracted one more time for 1 h in 1:5 ratio. Pooled supernatants were concentrated twofold using Smart Evaporator C1 (BioChromato, Fujisawa, Japan). All samples were filtered through syringe filter units Millex-LCR (Merck Millipore, Burlington, USA) with 0.45 μm pore size.

Samples were subjected to HPLC-MS analysis using Shim-pack HR-ODS column (2.21 × 150 mm, particle size 3 μm), Prominence HPLC chromatograph and LCMS-8040 mass spectrometer (Shimadzu, Kyoto, Japan) equipped with electrospray and set in MRM and ESI (–) mode. Injection volume was 10 μL. Eluents A and B were 0.1% water solution of formic acid and acetonitrile, respectively. Gradient profile was as follows: 0 min: 3% B; from 0 to 12 min linear gradient to 80% B; from 12.01 to 14 min: equilibration to the initial conditions of 3% B. The analytes were separated at a flow rate of 0.5 mL min^-1^ and column temperature of 40 °C. Parameters of mass spectrometry detection were as follows: desolvation line temperature 280 °C, heat block temperature 400 °C, nebulizing gas flow 3 L min^−1^, drying gas flow 15 L min^−1^. For phytohormones quantification, calibration curves for ABA (Olchemim, Olomouc, Czech Republic) and JA (Sigma-Aldrich, St. Louis, MO, USA) were constructed and corrected for [^2^H]_6_ABA internal standard.

### 4.6. Anatomical Study of Pea Stem

Cross sections of pea nodes and internodes were prepared from 8 mm segments embedded in paraffin. Cross sections of the segments were prepared with HM 325 rotary microtome (Thermo Fisher Scientific, Waltham, MA, USA) at thicknesses of 25 μm. The obtained cross sections were stained with Acridine Orange highlighting lignified tissues (cell walls of vascular bundles cells) in yellow to green. Acridine Orange has an excitation maximum of 490 nm and emission maximum of 535 nm. Microscopic pictures were performed by fluorescent microscopy under 10 × magnification with Olympus X71 inverted microscope and CPlanFN L 10×/0.3 objective lens (Olympus, Tokyo, Japan).

### 4.7. Statistical Analysis

Each series of experiments comprised at least eight replications; each replication was performed on a separate plant. Representative records obtained in individual measurements and mean values with standard errors are presented. Differences were examined with *t*-test and one-way ANOVA. Pearson correlation coefficient was used for correlation analysis.

## Supplementary Materials

The following are available online at https://www.mdpi.com/2223-7747/9/10/1364/s1, Figure S1: Simultaneous recording of transpiration using gas-exchange measurements and thermal imaging.
