# Supplementary material for: Spatial and Temporal Dynamics of Electrical and Photosynthetic Activity and the Content of Phytohormones Induced by Local Stimulation of Pea Plants
Source: Plants (Basel). 2020 Oct 15;9(10):1364. doi: 10.3390/plants9101364 (PMC7602463; doi:10.3390/plants9101364)
Supplement: Supplementary file 1 [file plants-09-01364-s001.pdf]

Supplementary Materials

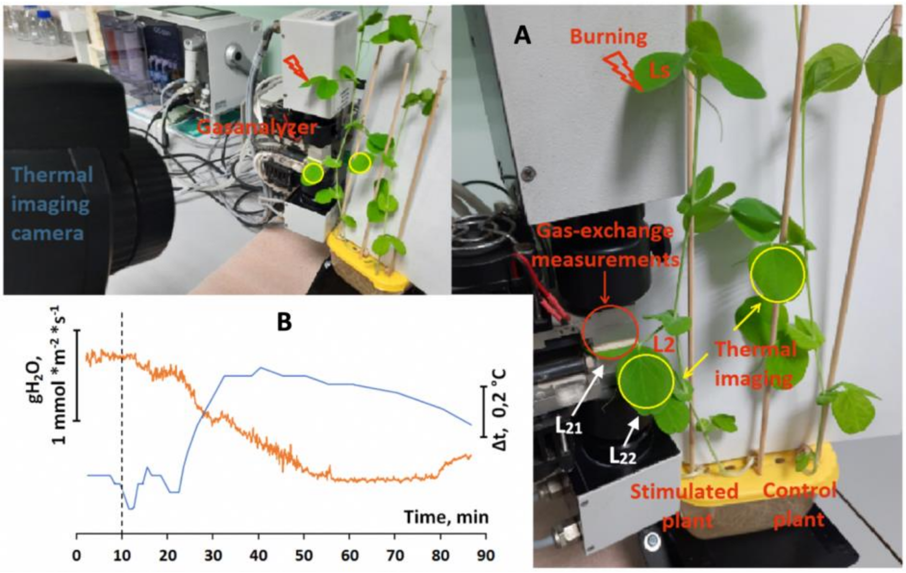

Figure S1. Simultaneous recording of transpiration using gas-exchange measurements and thermal imaging: (**a**) illustration of the experimental set-up; (**b**) dynamics of leaf temperature and transpiration induced by local burning. Transpiration was measured in the second leaf (L_2) below the stimulated leaf of pea. One of the two leaflets of the second leaf (L_2) was measured using a thermal imager (pointed with yellow circle), the other of the two leaflets was measured using gas analyzer. The temperature of the second leaf of control (unstimulated) plant was measured using thermal imaging. Δt is the temperature difference between the corresponding leaves of stimulated and control plants. Dashed line indicates the moment of stimulation. Variation potential (VP) generation was induced by burning tip of the upper fully developed leaf (L_S) with an open flame for 2–3 s. VP propagates in both leaflets of unstimulated leaf [22]. Leaflet (L_{21}) was measured using gas analyzer. Leaflet (L_{22}) of the second leaf (L_2) was measured using a thermal imager. Thermal images were taken together on control and stimulated plants. Registration of gas exchange parameters was carried out using an infrared gas analyzer GFS-3000, Heinz Walz GmbH, Effeltrich, Germany. The measurements were carried out under controlled conditions: an intensity of actinic blue light of 239 μmol m^{-2} s^{-1}, external CO_2 concentration of 360 μL L^{-1}, relative air humidity and temperature of about 70% and 23 °C, respectively. Measurements were performed on intact plants acclimated to a measuring system for 1.5 h.

Commented [M1]: There is no (a) and (b) in the figure.

Commented [M2]: There is no (a) and (b) in the figure.

Commented [M3]: Please check if it is right.

Commented [ML4R3]: It is right.
